# Development and Internal Validation of the Digital Health Readiness Questionnaire: Prospective Single-Center Survey Study

**DOI:** 10.2196/41615

**Published:** 2023-03-10

**Authors:** Martijn Scherrenberg, Maarten Falter, Toshiki Kaihara, Linqi Xu, Mayke van Leunen, Hareld Kemps, Hanne Kindermans, Paul Dendale

**Affiliations:** 1 Heart Centre Hasselt, Jessa Hospital Hasselt Belgium; 2 Faculty of Medicine and Life Sciences, Hasselt University Diepenbeek Belgium; 3 Faculty of Medicine and Health Sciences, Antwerp University Antwerp Belgium; 4 Department of Cardiology, Faculty of Medicine, Katholieke Universiteit Leuven Leuven Belgium; 5 Division of Cardiology, Department of Internal Medicine, St. Marianna University School of Medicine Kawasaki Japan; 6 School of Nursing, Jilin University Changchun China; 7 Department of Cardiology, Maxima Medical Centre Veldhoven Netherlands; 8 Department of Industrial Design, Eindhoven University of Technology Eindhoven Netherlands

**Keywords:** adherence, assessment, digital access, digital divide, digital health intervention, digital health, digital literacy, digital skills, health literacy, participation, telemedicine

## Abstract

**Background:**

While questionnaires for assessing digital literacy exist, there is still a need for an easy-to-use and implementable questionnaire for assessing digital readiness in a broader sense. Additionally, learnability should be assessed to identify those patients who need additional training to use digital tools in a health care setting.

**Objective:**

The aim of the development of the Digital Health Readiness Questionnaire (DHRQ) was to create a short, usable, and freely accessible questionnaire that was designed from a clinical practice perspective.

**Methods:**

It was a prospective single-center survey study conducted in Jessa Hospital Hasselt in Belgium. The questionnaire was developed with a panel of field experts with questions in following 5 categories: digital usage, digital skills, digital literacy, digital health literacy, and digital learnability. All participants who were visiting the cardiology department as patients between February 1, 2022, and June 1, 2022, were eligible for participation. Cronbach *α* and confirmatory factor analysis were performed.

**Results:**

A total number of 315 participants were included in this survey study, of which 118 (37.5%) were female. The mean age of the participants was 62.6 (SD 15.1) years. Cronbach *α* analysis yielded a score of >.7 in all domains of the DHRQ, which indicates acceptable internal consistency. The fit indices of the confirmatory factor analysis showed a reasonably good fit: standardized root-mean-square residual=0.065, root-mean-square error of approximation=0.098 (95% CI 0.09-0.106), Tucker-Lewis fit index=0.895, and comparative fit index=0.912.

**Conclusions:**

The DHRQ was developed as an easy-to-use, short questionnaire to assess the digital readiness of patients in a routine clinical setting. Initial validation demonstrates good internal consistency, and future research will be needed to externally validate the questionnaire. The DHRQ has the potential to be implemented as a useful tool to gain insight into the patients who are treated in a care pathway, tailor digital care pathways to different patient populations, and offer those with low digital readiness but high learnability appropriate education programs in order to let them take part in the digital pathways.

## Introduction

Digital health refers to the delivery or enhancement of health services and information via the internet and digital technologies [1]. After an initial phase in which multiple studies have already demonstrated the effectiveness of digital health technology in the field of medicine, more technologies are now being implemented as a standard of care [2]. With this evolution, caution should be taken not to create or increase a digital divide in which some people benefit from the new possibilities and others lag behind [3,4]. Similar to gaining insights about health literacy in the past, it is now of importance to assess the level of the digital access, skills, and literacy of the population that is being treated in each care pathway of health care.

The capability of a patient to work with new technology in a health care setting encompasses multiple elements. We propose using the term “digital readiness” to encompass the combination of digital access and usage, digital literacy, and digital health literacy.

One element, digital literacy, can be defined as “the extension of health literacy, but in the context of technology” [5]. It describes the ability to find, understand, and share content about health-related topics on digital media. These media include Wi-Fi, smartphones, and laptops, but also wearable technology like fitness trackers or smart watches [6].

Another element, digital health literacy, is described as the ability to seek, find, understand, and appraise health information from electronic sources and apply the knowledge gained to addressing or solving a health problem [7].

Tools to assess digital literacy or literacy already exist. Current tools have limitations, however, for their applicability in daily clinical practice by clinicians. The eHealth Literacy Scale (eHEALS) is a tool that was developed in 2006, and with ever-evolving technology, it does not grasp the current digital landscape anymore [8].

The eHealth Literacy Questionnaire (eHLQ), eHealth Literacy Framework, and eHealth Literacy Assessment toolkit [9,10] are important and recent tools in the landscape of digital health literacy research. The tools are excellent for digital literacy research, and they were developed to be used mainly by researchers, developers, and governments for the evaluation of digital technology. However, the frameworks and tools are lengthy and are therefore less suitable for implementation in routine clinical practice.

The aim of this study was first to develop an easy-to-use, short, and readily implementable questionnaire to assess the digital readiness of patients and perform an initial validation. Second, the aim was to incorporate questions about learnability to direct the future education of those with a low digital readiness.

## Methods

### Study Design and Study Population

This was a prospective single-center survey study conducted in Jessa Hospital Hasselt in Belgium. All participants who were visiting the cardiology department as patients between February 1, 2022, and June 1, 2022, were eligible for participation. The only exclusion criteria were insufficient understanding of Dutch and being younger than 18 years of age. A total of 500 questionnaires were distributed. The analysis only included Digital Health Readiness Questionnaire (DHRQ) that were fully completed.

In addition to completing the questionnaire, participants were asked to fill in their age, gender, and highest educational attainment. Furthermore, they were asked if they own a smartphone, tablet, laptop, internet connection at home, or smart watch. In some cases, the baseline characteristics were not fully completed however these cases were included in the analysis.

### Development of the Questionnaire

The DHRQ was developed based on (1) other questionnaires [9,10] and (2) the definition of readiness for digital health interventions by 2 medical doctors with extensive experience in digital health research. The objective of the DHRQ was to develop an instrument that captures digital skills, usage of digital technology, digital health literacy, and digital learnability, as well as digital literacy adapted for all digital health technologies such as smartphone apps, wearables, and internet-based interventions. Items for the questionnaire were simultaneously written in Dutch and English, as recommended by Eremenco, to avoid words or phrases that may be difficult to translate into other languages. Responses to the DHRQ were written following a 5-point Likert scale, in which correct answers were listed as strongly disagree, disagree, neutral, agree, and strongly agree, assigned a value of 1-5, respectively. The questionnaire was developed to connect a higher score to a better digital readiness. The questionnaire is provided in Multimedia Appendix 1.

The sum of the first 4 domains gives the total score on the DHRQ. The minimum score of the DHRQ is 15, and the maximum score of the DHRQ is 75. Additionally, a fifth domain called *digital learnability* is assessed. After completing the questionnaire, the result can be communicated as the total score with letters A-D from the digital learnability. In this way, the health care provider has an idea of the overall digital readiness and the potential learnability of the participant.

The DHRQ consists of 4 main domains, which is presented in Textbox 1.

**Domains of Digital Health Readiness Questionnaire (DHRQ).**

Digital usage

Digital usage assesses if the participants use devices such as smartphones, laptops, and wearables. This domain has 4 questions, with a minimum score of 4 and a maximum score of 20.

Digital skills

The domain digital skills assess the participants capabilities of performing different tasks on devices such as smartphones, laptops, and wearables. This domain has 5 questions, with a minimum score of 5 and a maximum score of 25.

Digital literacy

The digital literacy domain assesses how participants find, understand, and feel about information from web-based sources. This domain has 3 questions, with a minimum score of 3 and a maximum score of 15.

Digital health literacy

This domain assesses how participants interact with digital health tools and web-based health information. This domain has 3 questions, with a minimum score of 3 and a maximum score of 15. Total maximum score: 75

Additional category: digital learnability

This domain assesses the willingness and motivation of participants to improve their digital skills. This domain is not added to the total score of the DHRQ because it is not directly linked with the participants current digital health literacy level, but it aims to indicate that the literacy level can be increased with education. This domain has 5 questions, with a minimum score of 5 and a maximum score of 25. The score on the digital learnability domain is categorized into 4 groups: A (21-25), B (16-20), C (11-15), and D (5-10).

### Content Validity

An expert panel was consulted to ensure the content validity of the DHRQ; that is, 2 cardiologists, 2 nurses with experience in digital health, 3 biomedical experts in the field of digital health, a psychologist, and a patient. The expert panel received the initial draft questionnaire via mail and could make suggestions where needed. Their suggestions were used to optimize the DHRQ.

### Face Validity

Face validity was ascertained by presenting the questionnaire on paper to randomly selected participants in the hospital. They were asked to validate the DHRQ for question clarity and readability. An additional response process validation on paper was performed with 20 participants. These participants had to read all questions aloud and were recommended to think out loud to evaluate and ensure that all questions were correctly interpreted. The data of these participants were not used in the analysis. This process was solely performed to ensure that the questions were easy to understand. The face validity testing demonstrated that all questions were clear and easy to understand.

### Statistical Analysis

Data analysis was performed using SPSS (version 26; IBM Corp) at the Department of Medicine and Life Sciences (Hasselt University, Hasselt, Belgium). Categorical data were characterized by number and percentage. Continuous data were characterized by mean (SD) and median. The internal consistency of the 5 domains of the DHRQ was separately determined by calculating Cronbach *α* to assess the degree to which all the items of the DHRQ measure the same construct. A Cronbach *α* of >.7 was considered an adequate internal consistency for a questionnaire. Confirmatory factor analysis was performed based on the predefined domains. Model goodness-of-fit was assessed using the standardized root-mean-square residual, root-mean-square error of approximation, comparative fit index, and Tucker-Lewis fit index.

### Ethical Considerations

The protocol, informed consent form, and other study-related documents were approved by the Ethics Committee of Jessa Hospital Hasselt, Belgium, (file number 2021/140). All study participants provided written informed consent for their participation. All data and information that were collected during the study complied to the standards for protection of privacy in accordance with the General Data Protection Regulation.

The research institution provided insurance coverage for this study to all participants. No costs were charged to participants, and no payments were granted to participants in the context of this study.

## Results

A total of 315 participants were included in this survey study, of whom 118 (37.5%) were female. The mean age of the participants was 62.6 (SD 15.1) years. The minimum age was 21 years, and the maximum age was 97 years. In our sample, 251 (85.7%) participants owned a smartphone. Furthermore, most participants owned a tablet and Wi-Fi connection at home. Table 1 presents a more detailed description of the baseline characteristics.

Internal consistency is the degree in which a questionnaire evaluates the consistency per respondent between different items. Cronbach *α* analysis yielded a score of >.7 in all domains of the DHRQ, which indicates acceptable internal consistency. The domains digital literacy and digital learnability had the highest Cronbach α score. Only, the question about the usage of wearables skewed the results in the domain digital usage. Complete results can be found in Table 2.

**Figure 1:**
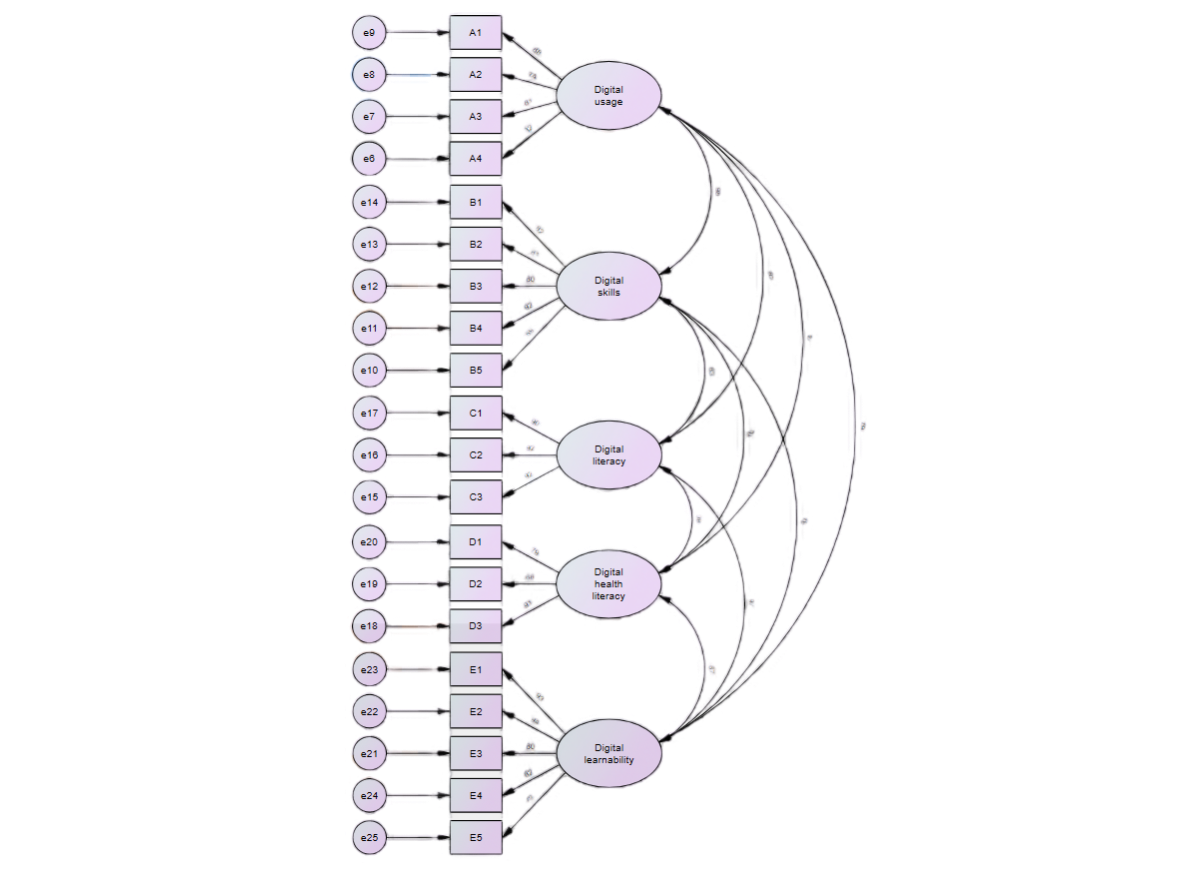
Framework for confirmatory factor analysis. e: error term.

Figure 1 gives an overview of the predefined 5-factor solution of DHRQ. Confirmatory factor analysis is a statistical technique used to verify the factor structure of a set of observed variables. The chi-square test for overall model fit was significant (*χ*^2^_160_=5590, *P*<.001). The fit indices showed a reasonably good fit: standardized root-mean-square residual=0.065, root-mean-square error of approximation=0.098 (95% CI 0.09-0.106), Tucker-Lewis fit index=0.895, and comparative fit index=0.912.

Table 3 gives an overview of the initial sensitivity analysis. A total of 15 patients did not respond to the question if they had a smart watch and 4 patients did not respond to the question if they had a smartphone. In this analysis, the total score on the questionnaire was compared to owning a smartphone and owning a smart watch, which was asked in separate questions to participants just prior to completing the DHRQ.

The theoretical maximum score of the DHRQ is 75. In our population, the mean score was 54.6 (73%, SD 15.2). Furthermore, the mean score for digital learnability was 18.8 (75%, SD 5.1). Interestingly, the DHRQ score and learnability were both significantly higher in the participants who owned a smartphone and participants who owned a smart watch.

Both age and an education level above secondary school were significant predictors of higher DHRQ scores. Higher age was associated with a lower DHRQ score, and higher education level was associated with a higher DHRQ score. Gender was not significantly associated with the total score. More information about the regression analysis can be found in Table 4.

**Table 1:** Baseline participant characteristics (N=315).

Variable		Values
Age (years), mean (SD)		62.7 (15.1)
Gender (female), n (%)		118 (37.5)
**Education level, n (%)**		
	Primary school	22 (6.9)
	Secondary school	141 (44.8)
	Professional bachelor	89 (28.3)
	Academic bachelor	50 (15.9)
Tablet ownership, n (%)		179 (56.8)
Wi-Fi connection at home, n (%)		279 (88.6)
Laptop or computer ownership, n (%)		273 (86.7)
Smart watch ownership, n (%)		89 (28.3)
Smartphone ownership, n (%)		255 (80.9)

**Table 2:** Cronbach *α* scores.

Variable	Score (N=315)
Digital usage	.735
Digital skills	.853
Digital literacy	.937
Digital health literacy	.837
Digital learnability	.925

**Table 3:** Outcome questionnaire and sensitivity analysis.

Variable	Total participants (N=315)	Participants with no smartphone (n=56)	Participants with a smartphone (n=255)	Participants with no smart watch (n=211)	Participants with a smart watch (n=89)	*P* value
Total score on the DHRQ^a^, mean (SD)	54.1 (18.6)	36.4 (17.1)	58 (12)	51.3 (15.5)	61.6 (12.8)	<.001
Total score on the DHRQ (%), mean (SD)	72.1 (24.8)	48.5 (22.8)	77.3 (16)	68.4 (20.6)	82.1 (17.1)	<.001
Digital learnability score, mean (SD)	15.4 (5.1)	13 (6.5)	19.8 (3.8)	18.1 (5.4)	20.3 (4)	<.001
Digital learnability (%), mean (SD)	61.6 (20.4)	52 (8.6)	79.2 (15.2)	72.4 (21.6)	81.2 (16)	<.001

^a^DHRQ: Digital Health Readiness Questionnaire.

**Table 4:** Multiple linear regression models to detect predictors of total Digital Health Readiness Questionnaire (DHRQ).

Variable	*B* (95% CI)	*β*	*P* value
Age	-0.433 (-0.534 to -0.332)	-0.423	<.001
Gender	-0.38 (-3.342 to 2.582)	-0.252	.80
Education level (professional bachelor or more)	6.946 (3.886 to 10.006)	4.467	<.001

## Discussion

In a health care setting in which digital technology has growing significance, it is increasingly important to better identify which patients need additional training or education and who might need additional follow-up when involved in a digital intervention.

### Principal Findings

In this study, a new questionnaire, the DHRQ was developed for assessing digital readiness and learnability in a health care setting. The results demonstrate a high internal consistency and a good sensitivity. The question about the usage of wearables skewed the results in the digital usage domain. Thus, while the use of wearables is growing in the current population, at this moment, it does not seem to correlate well with the other domains in the DHRQ. When comparing the results of the questionnaire in patients with a smartphone and a smart watch with those without any device, it is found that not only digital readiness is higher in the first group (as expected) but also learnability. Furthermore, lower age and higher education level were associated with a higher DHRQ score.

The DHRQ consists of 2 parts: the general DHRQ score and a score for the learnability of the participant. In this study, the learnability of all participants was high. The researchers believe that participants with a low DHRQ score should not per se be excluded from digital health interventions, as this would create and enhance an already existing “digital divide.” In contrast, the score should help identify those with lower scores in order to be able to provide additional training in the usage of digital health technologies. The learnability score could be used to assess their willingness and motivation to learn new technologies.

### Comparison to Prior Work

This questionnaire is not the first in the field of digital health literacy. In 2006, Norman and Skinner [8] developed an 8-item self-report eHEALS to measure digital skills, then mostly applicable to usage of the internet. The questionnaire is short and easy to use in clinical practice. However, technology has evolved in the last 16 years, and no questions incorporating current technology (smartphones and wearables) are part of the questionnaire. Also, authors have questioned if the questionnaire indeed measures what it intends to and have demonstrated only weak correlations with actual internet use, age, and education [11]. More recently, the eHealth Literacy Framework was defined, from which the eHLQ and eHealth Literacy Assessment tool kits are derived [9,10]. This framework and questionnaire assess 7 domains in a 57-item questionnaire. The questionnaire is extensive and has demonstrated robustness in its use. For routine clinical use, the questionnaire is lengthy; however, the authors state that it could possibly be of use to researchers, developers, and governments for the evaluation of digital technology.

### Strengths and Limitations

The DHRQ aims to bridge the gap between the easy-to-use but possibly outdated eHEALS questionnaire, the extensive but hard-to-implement eHLQ, and the incorporation of learnability, which immediately connects the outcome of the DHRQ to a possible intervention. To facilitate implementation, a web-based calculator was developed [12].

There are limitations to this study and the DHRQ. First, the DHRQ is an intentionally short questionnaire, which, therefore, cannot encompass all psychometric domains that are related to digital health literacy or readiness. Second, while internal consistency is good, only limited external validation has been performed by comparing to baseline demographics. In future studies, the DHRQ should be used in a real-life setting when implementing a digital care pathway to demonstrate a correlation with the uptake of the digital tools.

### Conclusions

To conclude, the DHRQ was developed as an easy-to-use, short questionnaire to assess the digital readiness of patients in a routine clinical setting. Initial validation demonstrates good internal consistency, and future research will be needed to externally validate the questionnaire. The DHRQ has the potential to be implemented as a useful tool to gain insight into the patients who are treated in a care pathway, tailor digital care pathways to different patient populations, and offer those with low digital readiness but high learnability appropriate education programs in order to let them take part in the digital pathways.

## Appendix

Multimedia Appendix 1 Digital Health Readiness Questionnaire.

## Abbreviations

DHRQ: Digital Health Readiness Questionnaire
eHEALS: eHealth Literacy Scale
eHLQ: eHealth Literacy Questionnaire

## Data Availability

Data are available on request from the corresponding author.

## References

[1] Saner H (2019). Digital health implementation: how to overcome the barriers?. Eur J Prev Cardiol.

[2] Scherrenberg M, Vangenechten G, Janssen A, Dendale P (2020). What is the Value of Digital Tools for Cardiovascular Patients?A Summary of a Comprehensive Review of the Evidence for (Cost-) Effectiveness for Prevention and Management of Cardiovascular Patients.

[3] Watts G (2020). COVID-19 and the digital divide in the UK. Lancet Digit Health.

[4] Falter M, Scherrenberg M, Kindermans H, Kizilkilic S, Kaihara T, Dendale P (2021). Willingness to participate in cardiac telerehabilitation: results from semi-structured interviews. Eur Heart J-Digit Health.

[5] Dunn P, Hazzard E (2019). Technology approaches to digital health literacy. Int J Cardiol.

[6] van der Vaart R, Drossaert C (2017). Development of the digital health literacy instrument: measuring a broad spectrum of health 1.0 and health 2.0 skills. J Med Internet Res.

[7] Richtering SS, Morris R, Soh S, Barker A, Bampi F, Neubeck L, Coorey G, Mulley J, Chalmers J, Usherwood T, Peiris D, Chow CK, Redfern J (2017). Examination of an eHealth literacy scale and a health literacy scale in a population with moderate to high cardiovascular risk: Rasch analyses. PLoS One.

[8] Norman CD, Skinner HA (2006). eHEALS: the eHealth literacy scale. J Med Internet Res.

[9] Karnoe A, Furstrand D, Christensen KB, Norgaard O, Kayser L (2018). Assessing competencies needed to engage with digital health services: development of the eHealth literacy assessment toolkit. J Med Internet Res.

[10] Kayser L, Karnoe A, Furstrand D, Batterham R, Christensen KB, Elsworth G, Osborne RH (2018). A multidimensional tool based on the eHealth literacy framework: development and initial validity testing of the eHealth literacy questionnaire (eHLQ). J Med Internet Res.

[11] van der Vaart R, van Deursen AJ, Drossaert CH, Taal E, van Dijk JA, van de Laar MA (2011). Does the eHealth literacy scale (eHEALS) measure what it intends to measure? Validation of a Dutch version of the eHEALS in two adult populations. J Med Internet Res.

[12] Online Digital Readiness Calculator.

