# Supplementary material for: Development and Internal Validation of the Digital Health Readiness Questionnaire: Prospective Single-Center Survey Study
Source: J Med Internet Res. 2023 Mar 10;25:e41615. doi: 10.2196/41615 (PMC10039407; doi:10.2196/41615)
Supplement: Multimedia Appendix 1 [file jmir_v25i1e41615_app1.docx]

MULTIMEDIA APPENDIX 1

A) Digital access

**I use the internet**

| Never / I don’t have access to the internet | Rarely | Sometimes | Often | Daily |
| --- | --- | --- | --- | --- |
| 1 | 2 | 3 | 4 | 5 |

**I use a computer and/or laptop**

| Never / I don’t have a computer and/or laptop | Rarely | Sometimes | Often | Daily |
| --- | --- | --- | --- | --- |
| 1 | 2 | 3 | 4 | 5 |

**I use a smartphone and/or tablet**

| Never / I don’t have a smartphone and/or tablet | Rarely | Sometimes | Often | Daily |
| --- | --- | --- | --- | --- |
| 1 | 2 | 3 | 4 | 5 |

**I use a wearable (fitness tracker, smartwatch, other)**

| Never / I don’t have any wearable | Rarely | Sometimes | Often | Daily |
| --- | --- | --- | --- | --- |
| 1 | 2 | 3 | 4 | 5 |

B) Usage of digital technology

**I am able to write and send an email independently.**

| Strongly disagree | Disagree | Neither agree nor disagree | Agree | Strongly agree |
| --- | --- | --- | --- | --- |
| 1 | 2 | 3 | 4 | 5 |

**I use social media such as Facebook, Instagram, other.**

| No | Rarely | Sometimes | Often | Daily |
| --- | --- | --- | --- | --- |
| 1 | 2 | 3 | 4 | 5 |

**I am able to perform videocalling.**

| Strongly disagree | Disagree | Neither agree nor disagree | Agree | Strongly agree |
| --- | --- | --- | --- | --- |
| 1 | 2 | 3 | 4 | 5 |

**I am able to take a picture and to send it to another person.**

| Strongly disagree | Disagree | Neither agree nor disagree | Agree | Strongly agree |
| --- | --- | --- | --- | --- |
| 1 | 2 | 3 | 4 | 5 |

**I am able to register and review my daily step count.**

| Strongly disagree | Disagree | Neither agree nor disagree | Agree | Strongly agree |
| --- | --- | --- | --- | --- |
| 1 | 2 | 3 | 4 | 5 |

C) Digital Literacy

*Being able to use digital technology to reach information.*

**I know how to find helpful and reliable information on the internet.**

| Strongly disagree | Disagree | Neither agree nor disagree | Agree | Strongly agree |
| --- | --- | --- | --- | --- |
| 1 | 2 | 3 | 4 | 5 |

**I feel safe when looking up information on the internet.**

| Strongly disagree | Disagree | Neither agree nor disagree | Agree | Strongly agree |
| --- | --- | --- | --- | --- |
| 1 | 2 | 3 | 4 | 5 |

**I feel in control when looking up information on the internet.**

| Strongly disagree | Disagree | Neither agree nor disagree | Agree | Strongly agree |
| --- | --- | --- | --- | --- |
| 1 | 2 | 3 | 4 | 5 |

D) Digital Health Literacy

*Being able to use digital technology to look up, use and work with health information.*

**I use the internet to find more information about my symptoms, health status and/or medication.**

| Strongly disagree | Disagree | Neither agree nor disagree | Agree | Strongly agree |
| --- | --- | --- | --- | --- |
| 1 | 2 | 3 | 4 | 5 |

**I use health-related applications to follow up my health status.**

| Strongly disagree | Disagree | Neither agree nor disagree | Agree | Strongly agree |
| --- | --- | --- | --- | --- |
| 1 | 2 | 3 | 4 | 5 |

**I am able to identify trustworthy, reliable health information on the internet.**

| Strongly disagree | Disagree | Neither agree nor disagree | Agree | Strongly agree |
| --- | --- | --- | --- | --- |
| 1 | 2 | 3 | 4 | 5 |

E) Learnability

*Motivation and interest to engage with new technology.*

**I am motivated to learn more about digital technology and how to use it myself.**

| Strongly disagree | Disagree | Neither agree nor disagree | Agree | Strongly agree |
| --- | --- | --- | --- | --- |
| 1 | 2 | 3 | 4 | 5 |

**I feel confident that I can learn more about digital technology and how to use it myself.**

| Strongly disagree | Disagree | Neither agree nor disagree | Agree | Strongly agree |
| --- | --- | --- | --- | --- |
| 1 | 2 | 3 | 4 | 5 |

**I believe that I will learn quickly when offered written information about digital technology.**

| Strongly disagree | Disagree | Neither agree nor disagree | Agree | Strongly agree |
| --- | --- | --- | --- | --- |
| 1 | 2 | 3 | 4 | 5 |

**I believe that I will learn quickly when offered personal guidance about digital technology.**

| Strongly disagree | Disagree | Neither agree nor disagree | Agree | Strongly agree |
| --- | --- | --- | --- | --- |
| 1 | 2 | 3 | 4 | 5 |

**I expect that learning digital skills can positively impact my health.**

| Strongly disagree | Disagree | Neither agree nor disagree | Agree | Strongly agree |
| --- | --- | --- | --- | --- |
| 1 | 2 | 3 | 4 | 5 |
